# Genetic variants help define the role of the MC4R C-terminus in signaling and cell surface stability

**DOI:** 10.1038/s41598-018-28758-3

**Published:** 2018-07-10

**Authors:** Bryn S. Moore, Tooraj Mirshahi

**Affiliations:** 1 Molecular and Functional Genomics, Weis Center for Research Geisinger Clinic, Danville, PA United States; 2 Geisinger Obesity Institute, Geisinger Clinic, Danville, PA United States

## Abstract

Screening 92,445 subjects in the Geisinger-Regeneron DiscovEHR cohort, we identified 5 patients heterozygous for nonsense mutations causing early terminations at Glu307 or Leu328 on the C-terminus of melanocortin 4 receptor (MC4R). Two Q307Ter carriers are severely obese (BMI > 40), while one is overweight (BMI > 25). One L328Ter carrier is overweight and the other is lean. Pedigree analysis for two Q307Ter carriers shows segregation of the variant with higher BMI. Functionally, MC4R(Q307Ter) eliminated receptor surface expression and signaling, while MC4R(L328Ter) functioned like the wild-type receptor. MC4R(Q307Ter) is therefore a loss of function (LOF) variant and the region between the two truncation sites identified in our patients is critical to MC4R function. Truncating MC4R at various C-terminal positions between these two variant sites, we find that cysteine318 sits at a critical junction for receptor trafficking and function. We show that MC4R is lipid modified at cysteine318 and cysteine319. Therefore, truncation early in the MC4R C-terminus results in haploinsufficiency in humans while truncation after the first lipid-modification site is well tolerated. MC4R haploinsufficiency clearly segregates with higher BMI; however, severe obesity is not fully penetrant even in MC4R LOF carriers, suggesting critical roles for environmental and lifestyle factors in MC4R monogenic obesity.

## Introduction

Obesity is a worldwide epidemic that contributes to comorbidities such as diabetes and cardiovascular disease^1^. Regulation of feeding and satiety, essential for maintaining healthy weight, occurs in the hypothalamus^2^. Satiety signals (α-MSH) released from pro-opiomelanocortin neurons and feeding signals (AgRP) released from agouti-related protein neurons are ligands for the melanocortin 4 receptor (MC4R)^3^. Since it receives both feeding and satiety signals, MC4R critically regulates feeding behavior and energy homeostasis.

MC4R is a member of the seven-transmembrane helices spanning domain containing G protein coupled receptor (GPCR) family. The C-terminus of MC4R contains a putative 8^th^ helical region typical of GPCRs. The 8^th^ helical region has been shown to play a regulatory role in several GPCRs including α_2B_-adrenergic receptor (AR) and angiotensin II type 1 A receptor (AT1R)^4^. Truncation of MC4R at K314 is a functional knockout in the rat^5^. An *in vitro* study has shown that truncation of MC4R after cysteine 319 results in a functional receptor^6^. It remains unclear why the region between K314 and C319, the ^315^EIICC^319^ region, encompassing part of the putative 8^th^ helical region of the MC4R C-terminus, is critical for receptor function.

Variants of MC4R have been reported in the ^315^EIICC^319^ region in obese individuals, specifically at amino acid 316 (I316S)^7^ and 317 (I317T)^8^. *In vitro*, EC_50_ for α-MSH activation of MC4R(I316S) and MC4R(I317T) are similar to the wild-type MC4R, however maximum receptor activity is reduced^7–9^. The I316S and I317T mutations of MC4R are less prevalent on the cell surface and are more highly modified by ubiquitin^10^, suggesting that not only trafficking to the membrane but length of residence for the receptor at the cell surface may be critical to MC4R function and its mechanism in obesity. In depictions of MC4R, two C-terminal cysteines, C318 and C319, are often shown with palmitoylation modifications^11,12^. However, aside from conservation analysis with the V2 vasopressin receptor, there is no direct evidence that MC4R is palmitoylated at either C318 or C319^6,13^.

Using whole exome sequencing data linked to longitudinal electronic health records from 92,445 participants in the Geisinger-Regeneron DiscovEHR project, we have found three participants that are heterozygous for truncation at Q307 and two that are heterozygous for truncation at L328 in MC4R. Q307Ter has been reported in 3 obese children and one adult^14–16^ and characterized as a non-functional receptor^15,17,18^. The L328Ter has only been reported in the Exac database^19^, but no phenotypic or functional data were reported. We test these truncations for cell surface expression and function and narrow down the essential amino acids in the C-terminus of MC4R to maintain function. Based on these functional data we have determined a critical role of the C-terminus of MC4R in its trafficking, function and demonstrate a mechanistic link to human obesity.

## Methods

### Study population, clinical variables and sequencing of MC4R

The research protocol was approved by the Geisinger Clinic Institutional Review Board, all participants provided written informed consent, and all experiments were performed in accordance with relevant guidelines and regulations. The authors did not have access to any identifying information for the participants. The human phenotype and genotype data in this study were all deidentified by a “data broker” who was not involved in the study before any analysis was performed. Genomic DNA was isolated from patients’ blood. Whole exome sequencing was performed in collaboration with Regeneron Genetics Center as previously described^20^. The sequence for MC4R for the five truncation variant carriers was confirmed using Sanger sequencing. De-identified clinical data was obtained from electronic health records (EHR). BMI measures were collected based on recorded values from each clinical visit for the patients.

### MC4R constructs

Untagged human MC4R or N-terminal 3x HA tagged MC4R in pcDNA3.1+ were purchased from cDNA.org. A bungarotoxin binding site (BBS) (MWRYYESSLEPYPD)^21^ epitope tag was added to the N-terminus of MC4R by PCR amplification and subcloned into pcDNA3.1 as described^22^. Individual mutations were made with the Quickchange site-directed mutagenesis kit (Stratagene). mCherry-2A-MC4R was constructed by subcloning mCherry with no stop codon 5′ to the 2A self-cleaving peptide sequence in pcDNA3.1^23^. MC4R and all the truncations were subcloned 3′ of 2A to create constructs that would express MC4R truncations and mCherry, ensuring that cells were indeed transfected. All constructs were confirmed by sequencing of the full-length clone.

### Cell culture

HEK293 cells (ATCC, Manassas, VA, USA) were cultured in MEM with 10% FBS at 37 °C and 5% CO_2_. For transient expression, cells were transfected with plasmids described above by Xtremegene (Roche, Indianapolis, IN, USA) and used two days post-transfection.

### Cell Surface Receptor Imaging

#### Bungarotoxin conjugated Texas Red

Cell surface imaging using fluorescently labeled Bungarotoxin (BTX) was carried out as described^22^. Bungarotoxin Binding Site (BBS) tagged MC4R constructs (BBS-MC4R) and Stargazin-CFP (to mark cell surface) were transfected into HEK293 cells on poly-L-lysine coated glass bottom fluorodishes (WPI, Sarasota, FL, USA). Cells were rinsed twice with physiological solution, which mimics the extracellular milieu (Imaging Low K), containing (in mmol/L): 25 HEPES, 114 NaCl, 2.2 KCl, 2 CaCl_2_, 2 MgCl_2_, 22 NaHCO_3_, 1.1 NaH_2_PO_4_, 2 glucose, pH 7.4, and labeled with cell impermeant 10 µg/mL Bungarotoxin (BTX) conjugated to Texas Red for 10–15 min at 4 °C and rinsed again in Low K solution. Live cells were imaged at room temperature within 15 minutes on an inverted Olympus Spinning Disc confocal microscope with Metamorph image acquisition software and processed with ImageJ. Images of single planes are shown in figures.

#### Bungarotoxin-biotin streptavidin-Qdots

To increase sensitivity of detection of the BBS-tagged cell surface receptors, we employed the following protocol to amplify the signal. Cells were prepared as above and labeled with 10 µg/mL BTX conjugated to Biotin for 10–15 min at 4 °C and rinsed in Low K solution thrice. Streptavidin conjugated Qdot-655 were added to cells for 10 min on ice. Cells were rinsed thrice with Imaging Low K. Live cells were imaged on a Zeiss LSM-710 confocal microscope and processed using Zen black software. Images of single planes are shown in figures.

### cAMP Microscopy Assay

cAMP production was measured using a Fluorescence Resonance Energy Transfer (FRET) assay as described^22^. Briefly, cells were transfected with mCherry-2A-MC4R constructs and Exchange Protein directly Activated by cAMP (EPAC2)-camps sensor^24^. Cells were imaged on an inverted Olympus Spinning Disc confocal microscope with Metamorph image acquisition software. The Metamorph software can record the position of multiple cells and the microscope is equipped with an automated stage, which allows for the repeated imaging of selected cells over a time-course. Cells expressing mCherry with similar intensities were selected and positions for each cell were recorded. CFP fluorescence and FRET between CFP and YFP were collected in cells expressing mCherry-2A-MC4R constructs identified by mCherry fluorescence. The FRET signal was normalized to the CFP signal for each cell. Baseline cAMP levels were established, and the same cells were exposed to 100 nmol/L of the MC4R agonist α-MSH, and then 100 µmol/L of the forskolin analog L-858051 (for maximum cAMP response). Each experiment was performed three times, with at least 10 cells imaged for each replicate. Data represents mean ± SEM from three independent experiments.

### cAMP pGlo Assay

HEK293 cells stably expressing pGloSensor-20F cAMP plasmid (Promega) under Hygromycin selection were transfected with mCherry-2A-MC4R or the truncation mutants in wells of a 6 well dish. The next day cells were re-plated in 12 wells of a white bottom 96 well dish (for cAMP assay) and wells of a clear bottom plate (to determine expression). The following day, the media was removed from the clear bottom dish and replaced with Imaging buffer and the fluorescence was read on a Spectramax M3 plate reader (Molecular Devices). Fluorescence from empty-vector transfected cells was subtracted from each group and then the values were normalized to the fluorescence reading for the wild-type mCherry-2A-MC4R from each experiment. Data is from 3 independent experiments (mean ± SEM). For the cAMP assay, the media was carefully removed from the white-bottom plate and replaced with media containing 2% GloSensor cAMP reagent (Promega) and incubated at 37 °C for 2 hours. Cells were stimulated with various concentrations of α-MSH for 10 min and then the luminescence was read. Basal cAMP luminescence was subtracted, and cAMP values plotted as a percentage of maximum wildtype cAMP. Data is from 3 independent experiments (mean ± SEM).

### S-acylation Assay

The S-acylation assay was performed as previously described^25^. Cells were transiently transfected with wild-type or mutant HA-MC4R. After two days, cells were rinsed with PBS and collected in hypotonic lysis buffer (10 mmol/L Tris, 5 mmol/L EDTA, pH7.4). A crude membrane extract was prepared by adding sucrose to 250 mM, lysed in a Dounce homogenizer and centrifuged to remove unlysed cells and cell debris. The lysate was ultracentrifuged at 60,000 rpm at 4 °C for 45 minutes and the pellet was resuspended in hypotonic lysis buffer (10 mM Tris pH7.4, 5 mM EDTA). The micro BCA protein assay (Thermo Scientific) was used to determine protein concentrations. The free (unmodified) thiol groups of equal amounts of proteins (2 mg) were blocked with 0.1% s-methylmethanethiosulfonate (MMTS), 100 mM HEPES, 1.0 mM EDTA, 2.5% SDS pH 7.5 and incubated at 40 °C for 10 mins with frequent vortexing. Three volumes of cold acetone were added, and the protein was precipitated at −20 °C for 20 mins and centrifuged to pellet the protein. The pellet was resuspended in 600 μL 100 mM HEPES, 1.0 mM EDTA, 1% SDS pH7.5 and split into 300 μL for the next step and 40 μL was saved as input control. 40 μL Thiopropyl sepharose beads (Sigma) and 40 μL of either 2 M NaCl (control) or 2 M hydroxylamine (pH 7.5, freshly prepared) were added to protein and were rotated overnight at 4 °C to remove S-acylation modifications. The beads were washed 5 × 2 mins and incubated in buffer with 50 mM DTT. Proteins were run on a 12% bis-tris gel and transferred to a nitrocellulose membrane. After blocking with 20 mL 5% milk and probing with an HRP-conjugated anti-HA antibody (Roche) (25 ng/mL in 20 mL 5% milk) the membrane was washed 3 × 10 mins in 20 mL TBS-T, incubated with 5 mL SuperSignal West Pico Chemiluminescent substrate (Thermo Scientific) and visualized with the Fujifilm LAS-4000 imaging system.

## Results

**Figure 1:**
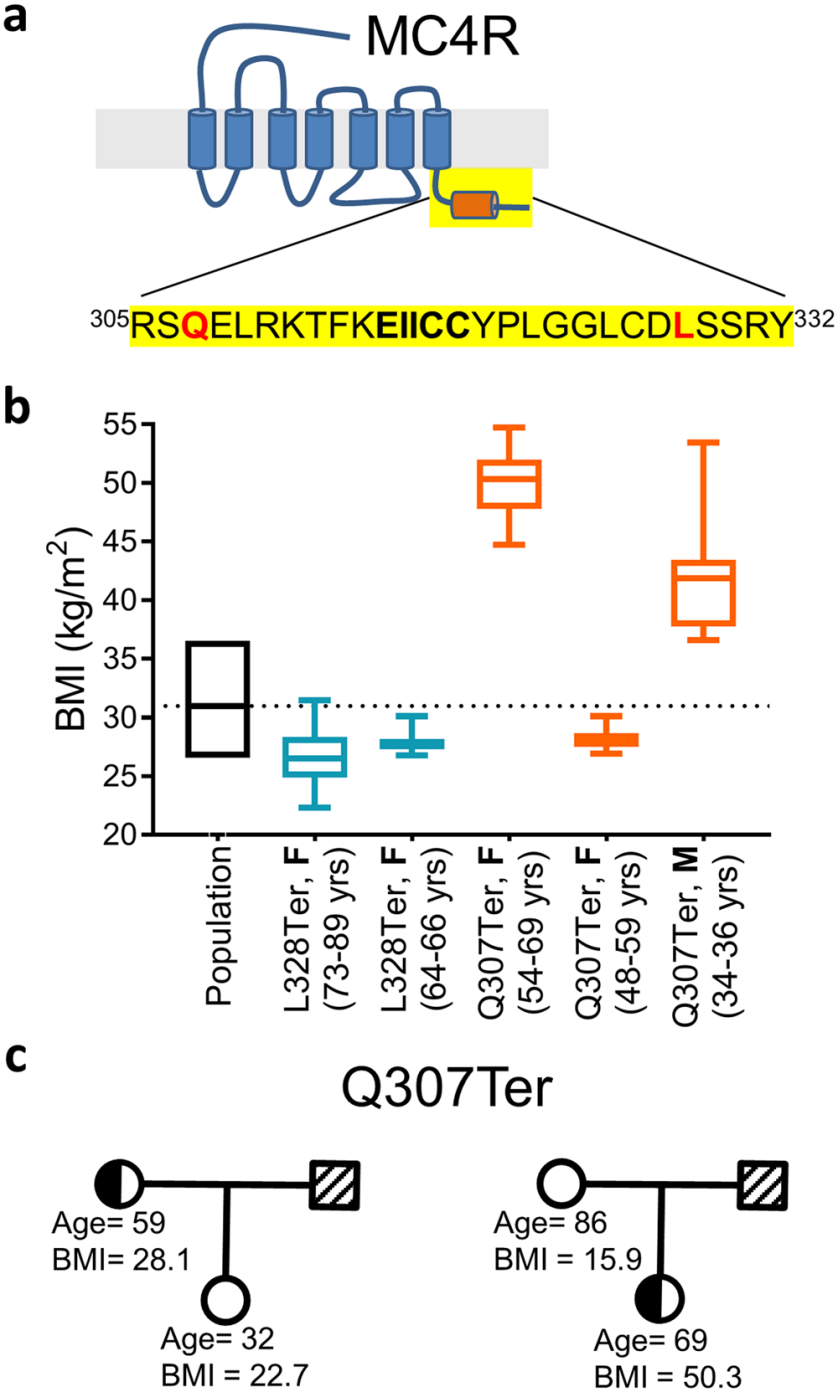
(**a**) Diagram of the C-terminus of MC4R (yellow) with the putative 8th helical region shown in orange. Early termination variants at Q307 and L328 (red) were found in our population. The ^315^EIICC^319^ region (bold) is part of the 8th helix on MC4R and may play a critical regulatory role in MC4R. (**b**) Median lifetime BMIs of non-carriers and individual patients with truncations at Q307 and L328 obtained from electronic health records and ages of the patients when the BMIs were recorded are reported. The follow-up for these patients was from 2–16 years. The box represents 25% and 75% quartiles and error bars are min and max for each patient. The dashed line is the median BMI for the cohort. The sex of each patient is denoted F for female or M for male. (**c**) Using available genetic data, we built limited pedigrees for two female (circle) carriers of Q307Ter. In both cases the Q307Ter variant co-segregates with higher BMI. Critically, the 59-year-old carrier, while not obese, had a BMI that was higher than her non-carrier daughter (28.1 vs 22.7 respectively).

Through whole exome sequencing of 92,445 participants in the MyCode cohort as part of the Geisinger-Regeneron DiscovEHR project, we found five participants heterozygous for early termination variants in the C-terminus of MC4R: three at Glutamine 307 (Q307Ter) (c.919 C > T) and two at Leucine 328 (L328Ter) (c.983 T > A) (Fig. 1a). L328Ter carriers have median BMI < 30, slightly below the median BMI for the cohort (~31). Two of the Q307Ter carriers are severely obese (BMI > 40), while one has a median BMI of 28.1 (Fig. 1b). Over half of the DiscovEHR participants have a 1st or 2nd degree relative in the cohort^26^. We have determined genetically related individuals and used these data to assemble *in silico* pedigrees when possible to look for co-segregation of genotypes with phenotypes^27^. We were able to construct limited pedigrees for two of the Q307Ter carriers with 1st degree relative non-carriers (Fig. 1c). One Q307Ter carrier has a median BMI of 50.3 whose non-carrier mother has a median BMI of 15.9. The father’s BMI and carrier status are unknown. The Q307Ter carrier that has a median BMI of 28.1 has a non-carrier daughter with a median BMI of 22.7. In both pedigrees, carrying the Q307Ter variant segregates with higher BMI.

**Figure 2:**
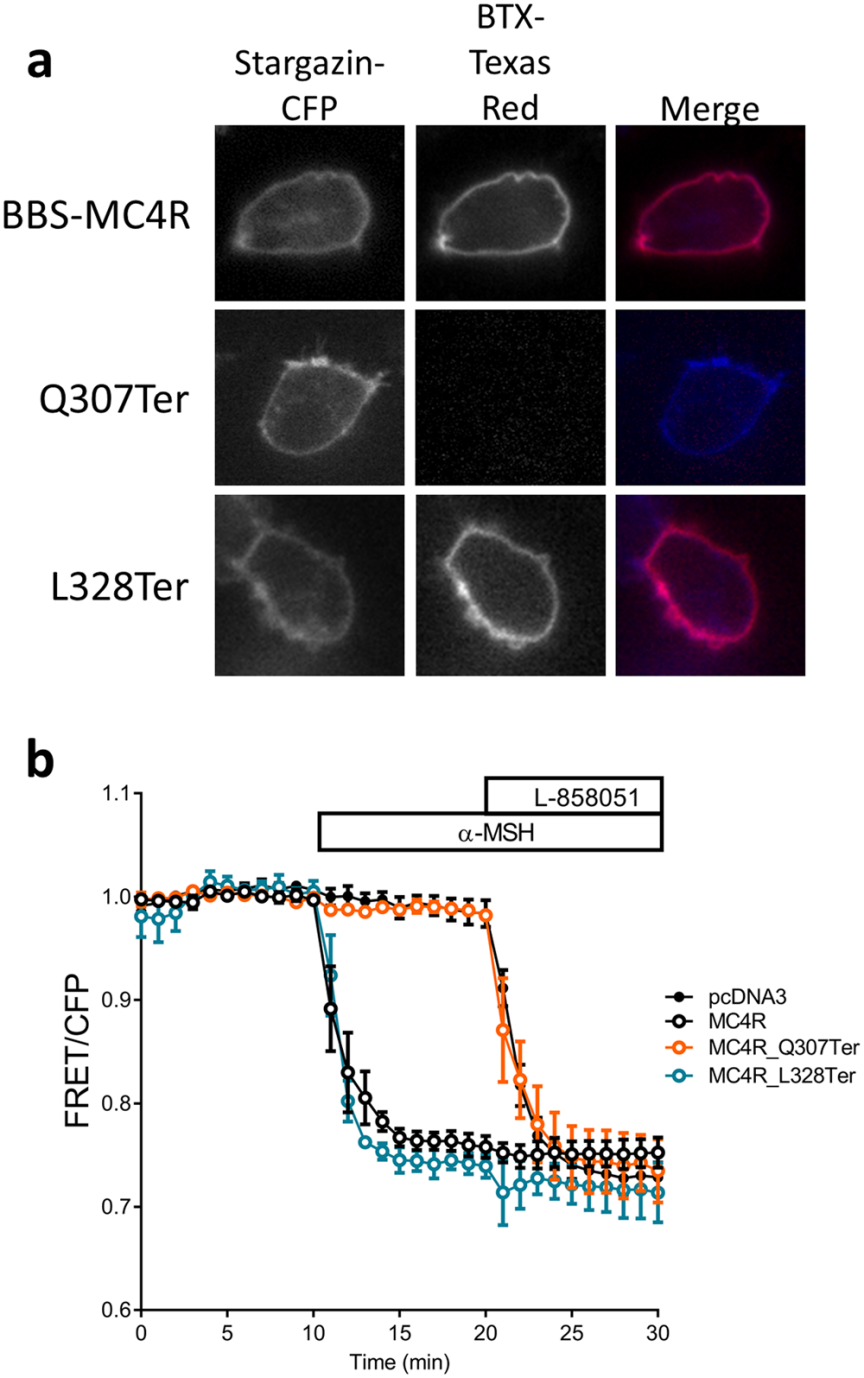
MC4R truncations identified in patients affect cell surface expression and signaling. (**a**) Cell surface localization, indicated by overlap with Stargazin-CFP which is a marker for the membrane, of truncated BBS-MC4R labeled with bungarotoxin-Texas Red expressed in HEK293 cells. (**b**) cAMP production depicted as reduced FRET in HEK293 cells expressing the EPAC sensor with either the wild-type or truncation mutants of MC4R found in our patients. Stimulation with MC4R agonist α-MSH (100 nM) results in robust changes in FRET for the WT and L328Ter while mock transfected cells and those expressing Q307Ter did not respond to α-MSH. The cyclase activator L-858051 (100 µM) was added at the end to activate maximum cAMP response. These data represent the mean ± S.E.M. of three independent replicate experiments where at least 10 cells were imaged per replicate.

Haploinsufficiency in MC4R is the most common form of monogenic obesity. Although bioinformatic prediction tools assigned the same pathogenicity (loss of function (LOF)) for both Q307Ter and L328Ter (Supplemental Table 1), based on previous studies we believed the Q307Ter variant will cause LOF and we hypothesized that the L328Ter variant will be functional. To test for cell surface expression, we tagged MC4R with a bungarotoxin binding sequence (BBS-MC4R) and made the two truncation mutations in BBS-MC4R^21,22^. We expressed BBS-MC4R, BBS-MC4R(Q307Ter) and BBS-MC4R(L328Ter) in HEK293 cells with Stargazin-CFP, which serves as a membrane marker, and used cell impermeant fluorescent bungarotoxin (BTX) to label cell surface expressed receptors in live cells. We did not observe significant levels of the Q307Ter mutant on the cell surface, while the L328Ter mutant showed similar surface expression to the wild-type receptor (Fig. 2a). We next tested agonist stimulated cAMP production using EPAC2-camps, a genetically encoded FRET-based cAMP sensor^24^. To ensure we are assaying cells that express each transfected mutant, we made a construct where MC4R is linked to mCherry with a self-cleaving 2A peptide. Agonist stimulation of BBS-MC4R and mCherry-2A-MC4R result in similar cAMP responses (Supplemental Fig. 1). Transfection of the mCherry-2A-MC4R construct results in 1:1 co-expression of MC4R and mCherry as they are cleaved after co-translation, therefore mCherry fluorescence allows us to identify cells that express the truncated receptor^23,28^. Cells transfected with Q307Ter mutant did not show any cAMP production in response to stimulation by the MC4R native agonist α-MSH, while L328Ter expressing cells showed robust cAMP response like those found in cells expressing wild-type MC4R (Fig. 2b). Given that truncation at Q307 did not traffic to the cell surface or function, while truncation at L328 was normal, we hypothesized that amino acids between these residues are critical to receptor trafficking and function.

**Figure 3:**
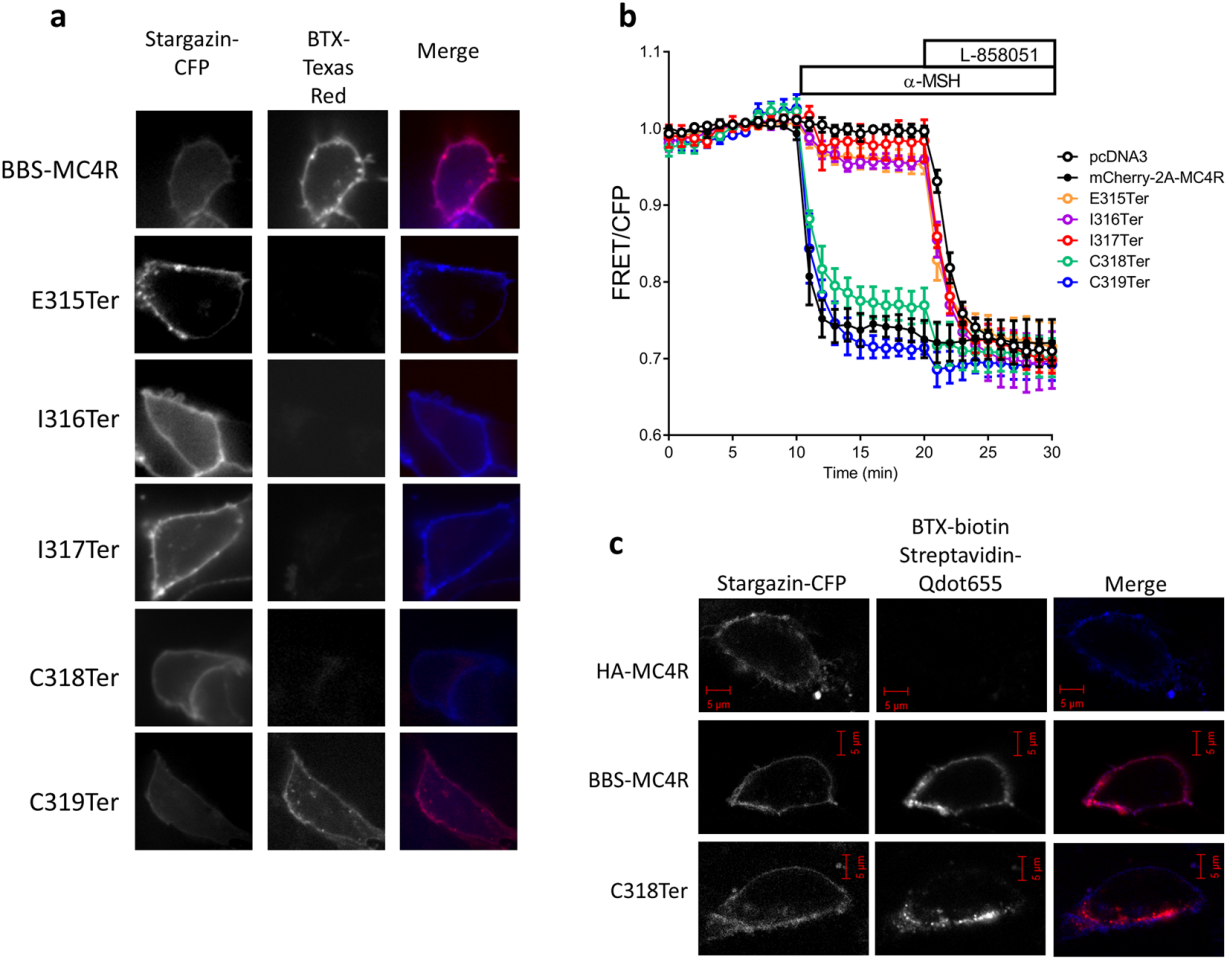
Truncation mutations in the ^315^EIICC^319^ region affect cell surface expression and signaling. (**a**) Cell surface localization, indicated by overlap with Stargazin-CFP which is a marker for the membrane, of bungarotoxin binding sequence (BBS)-tagged wild-type and truncated MC4R was observed using Texas Red conjugated bungarotoxin. WT and several truncation mutants are observed robustly on the cell surface, while truncations prior to C318 are not. Stargazin-CFP expression is used to label the plasma membrane. (**b**) cAMP production depicted as reduced FRET in HEK293 cells expressing the EPAC sensor as well as either the wild-type or C-terminal truncation mutants of MC4R. Stimulation with MC4R agonist α-MSH (100 nM) results in robust changes in FRET for the WT and truncations after C318, while mock transfected cells and those expressing truncations before C318 did not respond to α-MSH. The cyclase activator L-858051 (100 µM) was added at the end to activate maximum cAMP response. These data represent the mean ± S.E.M. of three independent replicate experiments where at least 10 cells were imaged per replicate. (**c**) Cell Surface expression of C318Ter truncation labeled with bungarotoxin-streptavidin and biotin-Qdot655 for brighter signal showing presence of C318Ter on the cell surface that seems to be more transient and therefore more difficult to capture during imaging.

Part of the region between the two patient truncation variants described above, encompasses the putative 8^th^ α-helix commonly found in GPCRs. This region is likely essential for proper localization to the cell surface, since truncation after C319 results in a functional receptor, but truncation at K314 impairs cell surface localization and signaling^5,6^ (Fig. 1a). We therefore asked which residues in the ^315^EIICC^319^ region of MC4R are essential for proper localization and function. We truncated BBS-MC4R at each position in this region, expressed them in HEK293 cells with Stargazin-CFP and used the BTX-assay to determine surface expression. We used a live cell approach because in addition to a yes, the receptor is on the surface or no, the receptor did not make it to the surface; live cell imaging gives us information regarding stability of receptors while on the cell surface. Truncation of MC4R before C318 impaired receptor cell surface localization; whereas, receptors truncated after C318 were expressed on the cell surface (Fig. 3a). We next used the EPAC2-camps FRET assay to assess the function of truncation mutants made in the mCherry-2A-MC4R construct. Truncating MC4R before C318 results in a receptor that does not signal (Fig. 3b). Truncation at, or after, C318 results in a functional receptor (Fig. 3b). To further support the functional findings in the FRET assay, we performed a pGLoSensor luciferase cAMP assay to determine the α-MSH induced cAMP production for these mutants compared to the WT receptor (Supplemental Fig. 2). The dose-response curves show that L328Ter and C319Ter function identically to the WT receptor with indistinguishable EC_50_ values (3.3, 2.7 and 2.5 nM respectively). Truncation mutants before position 318 did not function at any dose of α-MSH and the C318Ter was functional albeit with a higher EC_50_ compared to WT receptor (Supplemental Fig. 2).

We were surprised that stimulation of C318Ter generated a cAMP signal (Fig. 3b, Supplemental Fig. 2) given that we could not detect C318Ter on the cell surface (Fig. 3a). We hypothesized that C318Ter does make it to the cell surface, but at low abundance or with little stability. We tested this hypothesis using a higher sensitivity assay using BBS-MC4R constructs expressed in HEK293 cells and detected surface receptors using BTX conjugated to biotin and streptavidin labeled Q-dots to amplify the signal. C318Ter was detected on the cell surface; however, there was a significant amount of basal internalization when compared to the wild type (Fig. 3c). Therefore, the C318 residue is necessary for proper cell surface stability. Using the higher sensitivity labeling procedure, we repeated the surface expression experiments for all the BBS-tagged mutants shown in Figs 2a and 3a and corroborated those findings as well (Supplemental Fig. 3).

**Figure 4:**
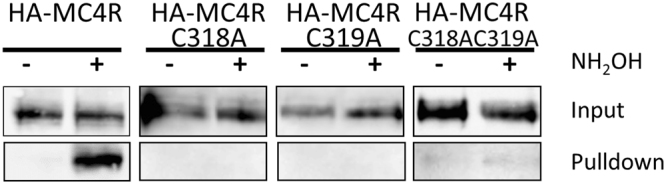
S-acylation assay of MC4R. HA-tagged wild-type (WT) MC4R, HA-MC4R(C318A), HA-MC4R(C319A) and HA-MC4R(C318AC319A) were pulled down and assessed for presence of acyl chains. WT MC4R was observed in the pulldown while the alanine mutants, to eliminate putative acyl-modification sites, were not, indicating not only acylation of MC4R but also the location of acylation. Paired pulldown of NH_2_OH-treated and -untreated samples for each mutant are run side-by-side (marked by black borders). Blot shown is representative of 3 similar experiments.

Conservation analysis of MC4R with the V2 Vasopressin receptor suggests that two cysteines at positions 318 and 319 on the C-terminus of MC4R are lipid modified; however, no empirical data exist for this lipid modification. Furthermore, the significance of this putative lipid modification is unclear. Using an S-acylation assay^25^ to pull down S-acylated proteins, we find that MC4R is lipid-modified (Fig. 4). Mutating C318, C319 or both to alanines prevented lipid modification (Fig. 4).

## Discussion

We have used our whole exome sequencing data in 92,445 subjects to assess the role of MC4R C-terminus in human obesity and on receptor function. Three variant carriers of Q307Ter were found in our Geisinger-Regeneron DiscovEHR populations. Two of the Q307Ter carriers are obese (BMI > 40) and one is overweight (BMI > 25). One of the L328Ter carriers is overweight while the other is lean. Limited *in silico* derived family pedigrees were assembled for two of the Q307Ter carriers, including the overweight subject. These pedigrees show segregation of the variant with higher BMI. At the molecular level, we show that the L328Ter functions like the wild-type receptor; while the Q307Ter is a LOF variant, with no observable receptor activity and failure to traffic to the cell surface. The role of MC4R in obesity is well established based on both human genetic data and animal knockout models. While MC4R-null mice are severely obese, MC4R heterozygous knockout mice show a milder obese phenotype. Therefore, MC4R haploinsufficiency leads to obesity in mice^29^. The Q307Ter is a LOF variant leading to MC4R haploinsufficiency and increased BMIs of carriers; whereas the L328Ter functions normally and carriers of this variant have BMIs well within the norm for our population.

Although mutations in MC4R lead to the most common form of monogenic obesity, not all variants are linked to obesity^30^. Even with the genetic burden of a MC4R LOF variant, the obesity phenotype is influenced by other genetic factors, both protective and deleterious, and environmental factors that contribute to BMI^16,31–33^. For instance, MC4R variant carriers who participated in lifestyle intervention, including physical exercise, nutrition education and behavior therapy, lost weight, but had difficulty maintaining weight loss^16^. Wild-type and MC4R heterozygous knockout mice show similar low levels of weight gain when fed a low-fat chow and heterozygous mice under these conditions are only mildly overweight^34^. We therefore speculate that the overweight, non-obese Q307Ter carrier may be protected from severe obesity by lifestyle or environmental factors. Similarly, the overweight L328Ter fits well with the norm of our cohort, which has a median BMI of ~31, where many genetic and environmental factors are likely at play to cause the overweight phenotype. These data support the critical role genetic and environment factors play for a complex phenotype like obesity, where a patient with a deleterious mutation in MC4R can still maintain a rather normal BMI.

The C-terminus of MC4R is essential for proper cell surface localization and signaling. Guided by patient variant data, we have established that truncation before C318 results in a receptor with improper localization and no signaling. Truncation at C318 interestingly leads to a receptor that can signal and can make it to the cell surface. However, the C318Ter mutant was only observed using a more sensitive cell surface labeling assay and it was rapidly internalized even in the absence of stimulus, suggesting C318Ter is unstable in the plasma membrane. Nevertheless, once a receptor reaches the cell surface, even briefly, it is exposed to the agonist. GPCR activity after internalization has been shown for several receptors^35^, suggesting that the C318ter may be briefly active at the cell surface and perhaps continue to signal even after internalization. Therefore, truncation of MC4R before C318 in patients results in a state of haploinsufficiency as demonstrated by Q307Ter. Whereas truncation after C318 has normal function and results in normal BMI as in the L328Ter carriers.

Although the role of lipid modification has been highlighted in other GPCRs, it had not been empirically tested in MC4R. S-acylation can incorporate palmitate, stearate or oleate fatty acids. However, of all S-acylated platelet proteins 74% of the modifications were palmitoylations^36^. We showed that MC4R is S-acylated on the C-terminus and that removing cysteine at positions 318 and 319 eliminates acylation. We speculate that cell surface stability is aided by S-acylation modification at C318/C319, given that the C318Ter mutant was only briefly present on the cell surface. Nevertheless, even the C318Ter mutant was able to signal properly to the cAMP pathway. Acylation, most likely palmitoylation, may be a mechanism by which MC4R regulates its residency on the cell’s surface to perhaps control the duration of signaling. Determining the physiological significance and mechanism of MC4R S-acylation and whether it can be manipulated to modify receptor activity will provide new insights into controlling feeding and satiety.

With increasing availability of population genetic data and in the era of precision medicine, the genetics of individual subjects are being used for their specific care and treatment. However, while population-level data based on genetic findings have established associations as well as cause-effect relationships for many genes and diseases, the role that functional assessment of genetic variants plays cannot be minimized. Bioinformatic prediction tools, commonly used for rapid classification of genetic variants, often use conservation or other generalizable data to call a variant pathogenic or benign. As such, both variants we identified, Q307Ter and L328Ter have the same predicted effect according to the Variant Effect Predictor on Ensembl^37^ and most other bioinformatic prediction tools (Supplemental Table 1). Our data clearly show the MC4R truncation at position 328 was not a LOF while the truncation at position 307 is a LOF. Our findings highlight the necessary role functional assessment plays in determining the pathogenicity of variants in human disease. Finally, even though 3 patients had the same Q307Ter LOF variant, the phenotype in one patient was much milder than the other two, indicating the critical role environmental factors and individuals’ lifestyles can play in a complex phenotype such as obesity.

## Electronic supplementary material

Supplementary Information
