# Supplementary material for: Genetic variants help define the role of the MC4R C-terminus in signaling and cell surface stability
Source: Sci Rep. 2018 Jul 10;8:10397. doi: 10.1038/s41598-018-28758-3 (PMC6039487; doi:10.1038/s41598-018-28758-3)

Genetic variants help define the role of the MC4R C-terminus in signaling and cell surface stability.

Bryn S. Moore[1] and Tooraj Mirshahi[1,2]

Molecular and Functional Genomics, Weis Center for Research[1] and Geisinger Obesity Institute[2], Geisinger Clinic, Danville, PA, United States

Supplemental Material

Supplemental Table 1. Table of Variant Effect Predictor on Ensembl[36] bioinformatic prediction algorithm results for the Q307Ter and L328Ter variant. Several common prediction algorithms failed to provide a result (e.g. Polyphen), but most of the others predicted that both Q307Ter and L328Ter would be loss of function, with nearly identical scores.

Supplemental Figure 1. cAMP production depicted as reduced FRET in HEK293 cells expressing the EPAC sensor as well as either BBS-MC4R or mCherry-2A-MC4R. Stimulation with MC4R agonist α-MSH (100 nM) results in robust and similar changes in FRET. The cyclase activator L-858051 (100 µM) was added at the end to activate maximum cAMP response.

Supplemental Figure 2. cAMP production in response to various doses of α-MSH measured using the pGlow plate-based assay in HEK293 cells expressing the pGloSensor-20F cAMP plasmid as well as mCherry-2A-MC4R and various MC4R mutants as depicted in the legend. Stimulation by α-MSH for WT-MC4R, L328Ter, and C319 show similar EC_{50} and maximum response, while truncation preceding position 317 did not produce cAMP at any dose. C318ter was activated by α-MSH however with a higher EC_{50} (25.0 nM). Bottom bar graph shows

similar expression for the various MC4R constructs measured based on fluorescence from the co-translated mCherry.

Supplemental Figure 3. High sensitivity cell surface expression of truncation mutations of BBS-MC4R labeled with bungarotoxin-streptavidin and biotin-Qdot605 for brighter signal showing only truncations at or after C318 are on the cell surface. Stargazin-CFP expression is used to label the plasma membrane.

Supplemental Table 1.

	Variant	Variant	Explaination of the score	Website
Uploaded variant	18_60371431_G/A	18_60371367_A/T		
Location	18:60371431-60371431	18:60371367-60371367		
Allele	A	T		
Protein change	Q307Ter	L328Ter		
Consequence	stop_gained	stop_gained		
Impact	HIGH	HIGH		
Symbol	MC4R	MC4R		
Gene	ENSG00000166603	ENSG00000166603		
Feature type	Transcript	Transcript		
Feature	ENST00000299766	ENST00000299766		
Biotype	protein_coding	protein_coding		
Exon	1/1	1/1		
cDNA position	1345	1409		
CDS position	919	983		
Protein position	307	328		
Amino acids	Q/*	L/*		
Codons	CAA/TAA	TTG/TAG		
Existing variant	rs745973015, CM091289	rs751914635		
Feature strand	-1	-1		
Symbol source	HGNC	HGNC		
HGNC ID	HGNC:6932	HGNC:6932		
LoFtool[(p)]	0.771	0.771	A percential score for gene intolerance to functional change. The lower the score the higher gene intolerance to functional change.	DOI:10.1093/bioinformatics/btv602
CADD raw[(p)]	13.755027	13.431919	A larger the score the more likely a functional affect. Range of score is -7.54 to 35.79	http://cadd.gs.washington.edu/
DANN score[(p)]	0.997152468	0.992831018	A larger the score the more likely a functional affect. Range of score is 0 to 1	https://cbcl.ics.uci.edu/public_data/DANN/
Eigen-raw[(p)]	1.309343485	0.74704274	A functional prediction score based on conservation,allele frequencies, and deleteriousness prediction using an unsupervised learning method	http://www.columbia.edu/~ii2135/eigen.html
GenoCanyon score[(p)]	1	0.999853474	A functional prediction score based on conservation and biochemical annotations using an unsupervised statistical learning	http://genocanyon.med.yale.edu/index.html
LRT pred[(p)]	D	N	D(eleterious), N(eutral) or U(nknown)	http://www.genetics.wustl.edu/jflab/lrt_query.html
MutationTaster pred[(p)]	D	D	Alterations causing a premature termination codon and ultimately leading to nonsense-mediated mRNA deday (NMD) are automatically assigned the 'disease causing' status.	http://www.mutationtaster.org/
SiPhy 29way logOdds[(p)]	18.1147	13.8738	The larger the score, the more conserved the site. Scores range from 0-37.9718	http://www.broadinstitute.org/mammals/2x/siphy_hg19/
fathmm-MKL coding score[(p)]	0.99031	0.96959	Scores range from 0 to 1. Scores >0.5 are predicted to be deleterious	http://fathmm.biocompute.org.uk/fathmmMKL.htm

Supplemental Figure 1.

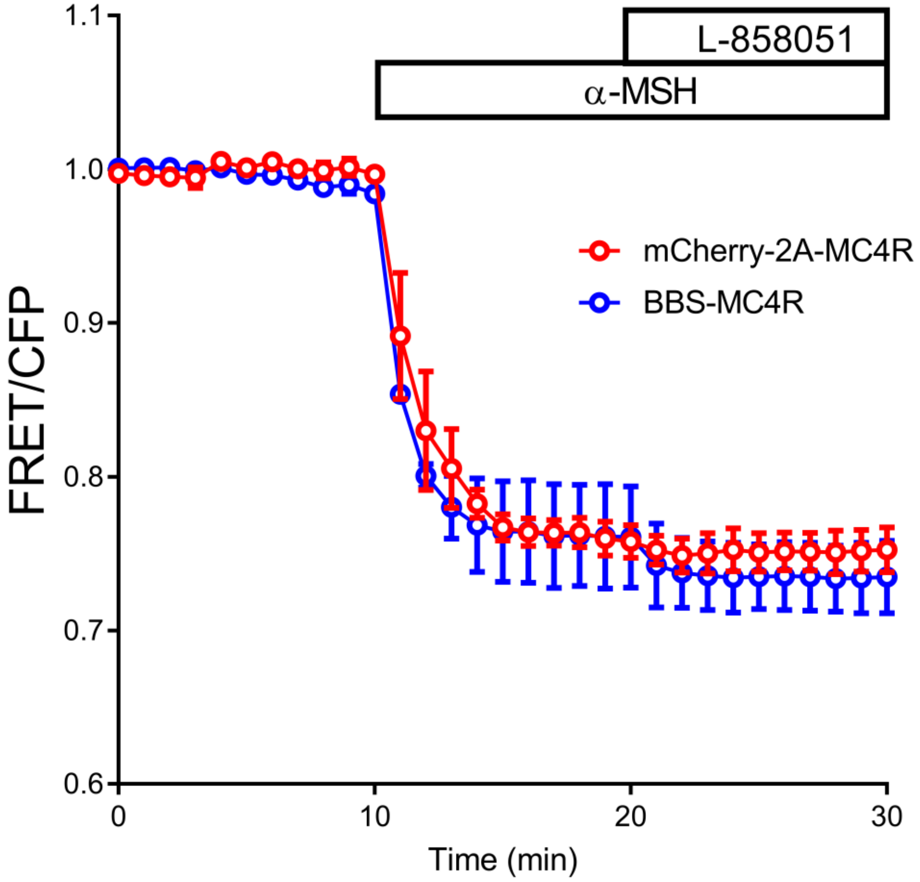

Supplemental Figure 2.

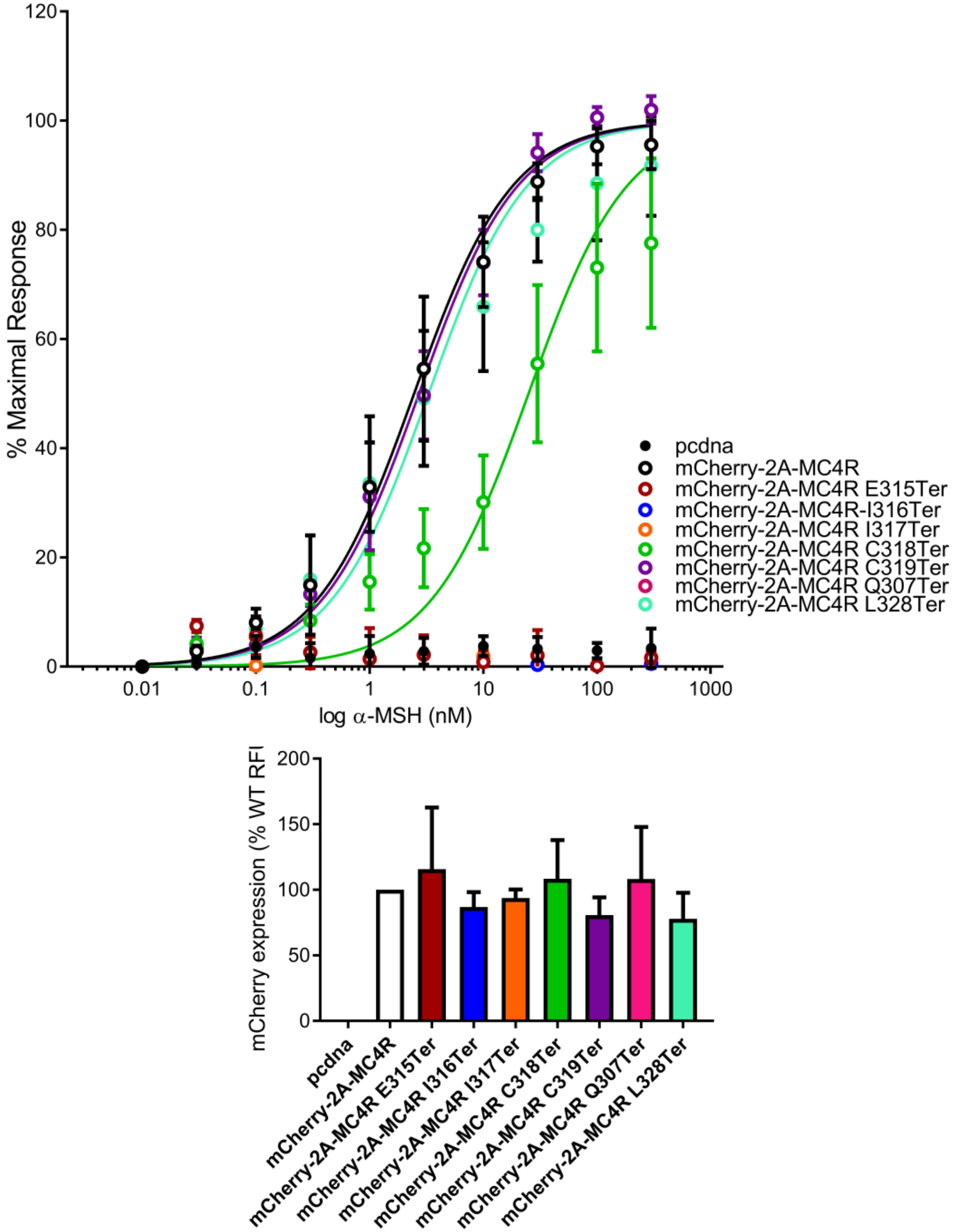

Supplemental Figure 3.

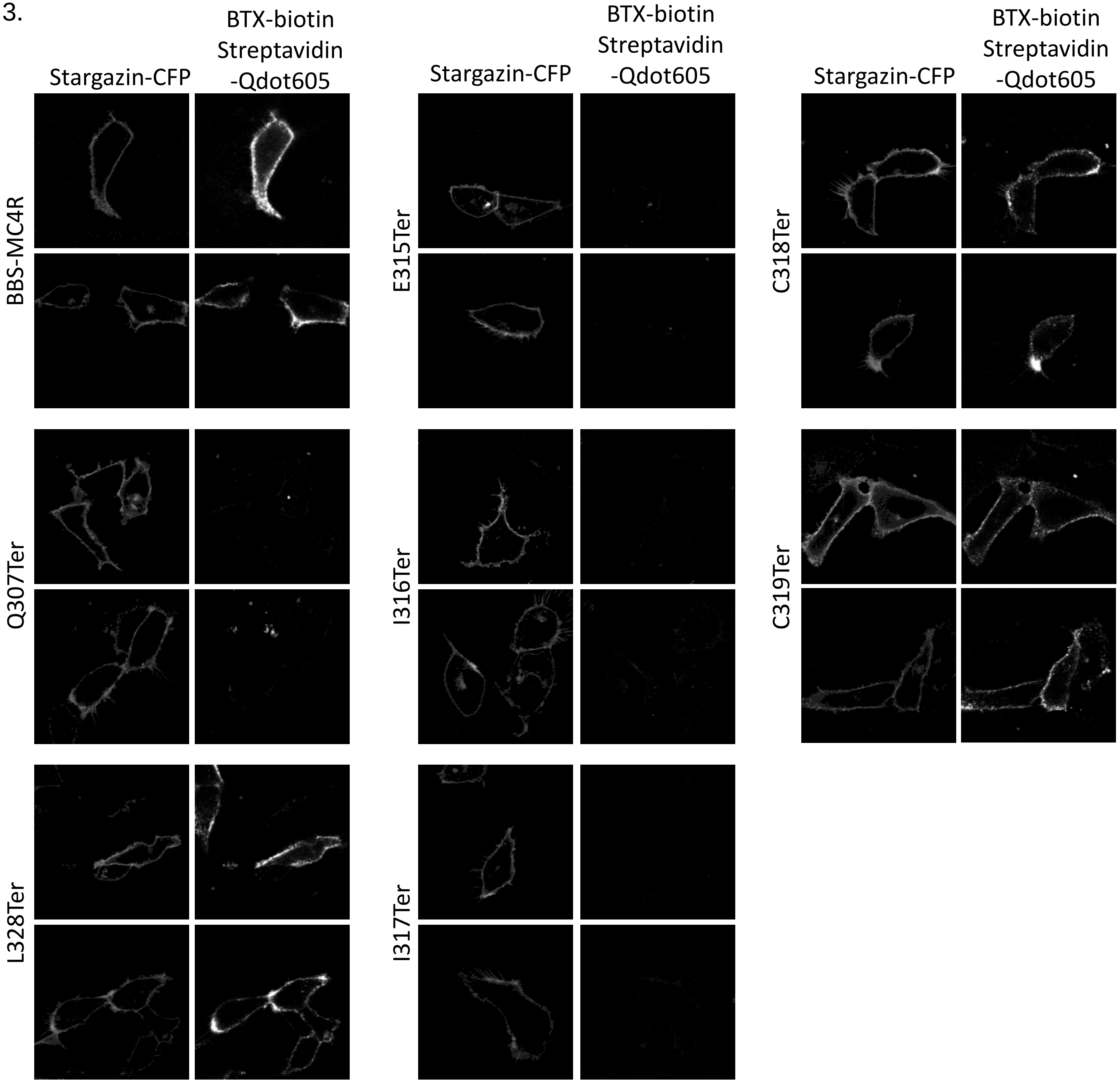

Supplement: Supplementary file 1 — Supplementary Information [file 41598_2018_28758_MOESM1_ESM.pdf]
